# DNA Materials Assembled from One DNA Strand

**DOI:** 10.3390/ijms24098177

**Published:** 2023-05-03

**Authors:** Jiezhong Shi, Ben Zhang, Tianyi Zheng, Tong Zhou, Min Guo, Ying Wang, Yuanchen Dong

**Affiliations:** 1 Sinopec Beijing Research Institute of Chemical Industry, Beijing 100013, China; 2 CAS Key Laboratory of Colloid Interface and Chemical Thermodynamics, Beijing National Laboratory for Molecular Sciences, Institute of Chemistry, Chinese Academy of Sciences, Beijing 100190, China; 3 University of Chinese Academy of Sciences, Beijing 100049, China

**Keywords:** DNA materials, one DNA strand, DNA assembly, palindrome, nanostructure

## Abstract

Due to the specific base-pairing recognition, clear nanostructure, programmable sequence and responsiveness of the DNA molecule, DNA materials have attracted extensive attention and been widely used in controlled release, drug delivery and tissue engineering. Generally, the strategies for preparing DNA materials are based on the assembly of multiple DNA strands. The construction of DNA materials using only one DNA strand can not only save time and cost, but also avoid defects in final assemblies generated by the inaccuracy of DNA ratios, which potentially promote the large-scale production and practical application of DNA materials. In order to use one DNA strand to form assemblies, the sequences have to be palindromes with lengths that need to be controlled carefully. In this review, we introduced the development of DNA assembly and mainly summarized current reported materials formed by one DNA strand. We also discussed the principle for the construction of DNA materials using one DNA strand.

## 1. Introduction

As the carrier of genetic information [1], DNA has become the center of biological research and attracted widespread attention in recent decades [2]. In 1982, inspired by the construction of branched structures using the Holliday Junction model in organisms [3], Professor Seeman firstly proposed the concept of DNA assembly and mentioned that DNA could be utilized as bricks to prepare 3D nanostructures [4], which paved the development of a new field, DNA nanotechnology [5]. The DNA molecule exhibits many excellent properties, such as the specific base-paring recognition, programmable sequences, clear structures in nanoscale and responsiveness of functional sequences [6], making it an ideal material for self-assembly [7]. To date, a large number of materials have been fabricated based on DNA assembly, such as nanostructures [8], microparticles [9] and hydrogels [10], which have been widely used in drug delivery [11], controlled release [12] and tissue engineering [13].

Currently, tile assembly [14] and DNA origami [15] are two fundamental strategies for DNA assembly, in which multiple DNA strands are required to form DNA materials. This process makes the synthesis and assembly of DNA strands time consuming and expensive. More importantly, the molar ratios of DNA strands have to be strictly controlled in the assembly process to ensure the formation of intact structures. Thus, high standards and requirements are necessary for assembly operation and DNA purities.

In 2006, Mao et al. firstly tried to prepare large nanostructures using just one DNA strand and successfully constructed DNA nanotubes [16]. This approach greatly saved time and cost for DNA synthesis and assembly, and avoided the difficulties of controlling the molar ratios of different DNA strands, providing a new platform for DNA assembly and potentially accelerating the large-scale production and practical application of DNA materials. Here, in this review, following a brief overview of the development of DNA assembly, we firstly reviewed current reported materials from 1D to 3D assembled by one DNA strand, and then summarized the principle for the construction of DNA materials using one DNA strand. We also discussed some challenges in this field and proposed the perspectives for DNA materials.

## 2. The Development of DNA Assembly

**Figure 1:**
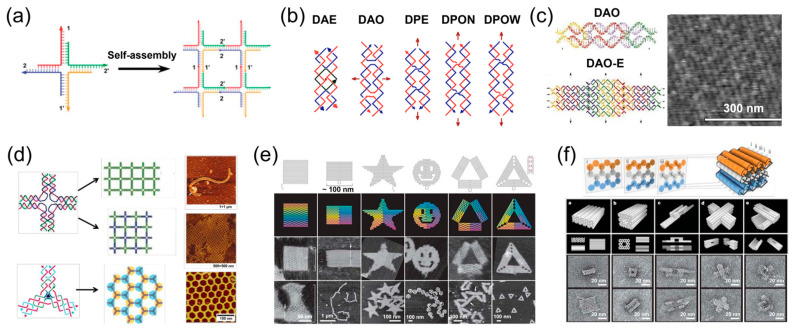
Nanostructures formed by DNA assembly. (**a**) Assembly of four-arm DNA junctions [4]; (**b**) structure of Five classic DX models [17]; (**c**) AFM image of assembled 2D structures [18]; (**d**) formation of nanoribbons and nanogrids [19]; (**e**) 2D assemblies formed by DNA origami [22]; (**f**) 3D nanostructures formed by DNA origami [23].

As mentioned above, the development of DNA assembly could be traced back to 1982, in which Professor Seeman proposed a four-arm DNA junction [4]. As shown in Figure 1a, this structure was composed of four DNA single strands. The sticky ends on each arm were able to hybridize with other four-arm junctions and potentially assembled into 2D DNA structures. Later, Seeman et al. designed a series of more stable double-crossover (DX) structures which contained two DNA double helix structures and exhibited stronger rigidity [17]. As illustrated in Figure 1b, five classic DX models were systematically studied and the design of these models was optimized, providing a basic platform for the construction of complex nanostructures. Due to the limitation of technology, Seeman et al. did not observe the assembly structures under the microscope. Only gel electrophoresis was utilized to verify the formation of large assemblies. In 1988, Winfree et al. successfully observed the structure assembled by DX motifs under AFM (atomic force microscope) for the first time (Figure 1c) [18]. Subsequently, Yan et al. designed a novel four-arm junction by introducing the DX structure in each arm [19]. As illustrated in Figure 1d, the designed junction exhibited higher stability and could assemble into nanoribbons and nanogrids. Based on the four-arm junction, Mao et al. designed three-arm and six-arm junctions. By adjusting the concentration, curvature and rigidity of the junction, many 3D structures were constructed, such as tetrahedron, dodecahedron and icosahedron [20,21]. These structures were also characterized by AFM and Cryo-TEM (cryogenic transmission electron microscopy). In order to simplify the system, Mao et al. also fabricated assemblies using only one DNA strand [16], which provided a new platform for DNA assembly.

In the aforementioned systems, DNA strands firstly assemble into small tiles, and then form complex assemblies by the hybridization of the sticky ends of these tiles. This strategy is called tile assembly [14], and requires precise design of DNA sequences and the efficiency of DNA assembly would restrict the formation of large nanostructures. In 2006, Professor Rothemund developed a novel assembly strategy called DNA origami [22]. As shown in Figure 1e, in this strategy, a long DNA strand (scaffold strand) was arranged into the defined shape, and then a large number of short DNA strands (staple strands) were introduced to fix the shape through the formation of double-crossover structures. This is a more universal strategy, and nanostructures with arbitrary shapes could be designed with the assistance of computer software. Later, Shih et al. extended this strategy to build custom 3D structures by changing the base number of staple strands to control the angle between the double helix (Figure 1f) [23]. Yan et al. also prepared 3D spherical shells, ellipsoidal shells, and nanoflasks through the precise control of curvature [24].

**Figure 2:**
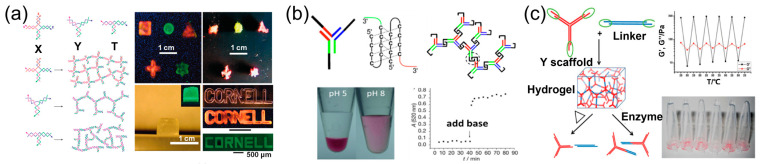
Hydrogels formed by DNA assembly. (**a**) DNA hydrogels crosslinked by DNA ligase [25]; (**b**) pH-responsive DNA supramolecular hydrogels [26]; (**c**) DNA supramolecular hydrogels with thermal and enzymatic responsiveness [27].

In addition to the nanostructures, DNA has also been used to fabricate the hydrogels. In 2006, Luo et al. constructed three-arm and four-arm branched building blocks by DNA self-assembly and connected these building blocks together through the formation of phosphodiester bonds using DNA ligase to fabricate DNA hydrogels (Figure 2a) [25]. This hydrogel has been tested for potential application in drug loading, cell culture and in vitro expression. In 2009, Liu et al. proposed pure DNA supramolecular hydrogels based on DNA assembly [26]. As shown in Figure 2b, the building block of this hydrogel was a Y-shaped assembly hybridized by three short DNA strands with half i-motif sequence at the end. In acidic conditions, the Y-shaped assembly could be crosslinked through the formation of i-motif structures, leading to the construction of hydrogel. The hydrogel could be converted into solution state with the increase in pH. It is notable that the hydrogel network was composed of rigid DNA double strands and i-motif structures that could not collapse, resulting in the absence of small pores under a certain size. Therefore, the hydrogel had good permeability and the large molecules can easily diffuse in the hydrogel network. Later, Liu et al. designed a DNA double-strand structure with sticky ends as the linker for Y-shaped assembly and successfully prepared stable DNA supramolecular hydrogels under physiological conditions (Figure 2c) [27]. Taking advantage of the rapid formation, high permeability and dual responsiveness, this hydrogel has been widely used in controlled release [28], tissue engineering [29] and 3D printing [30].

With the continuous development of DNA assembly, a variety of sophisticated structures and functional materials have been prepared to date [31,32,33,34,35,36,37,38,39,40,41,42,43,44], opening up a new frontier for macromolecular self-assembly.

## 3. The 1D Materials Assembled by One DNA Strand

The construction of 1D materials using one DNA strand is the most fundamental, and subsequent preparation of 2D and 3D materials are all based on this.

**Figure 3:**
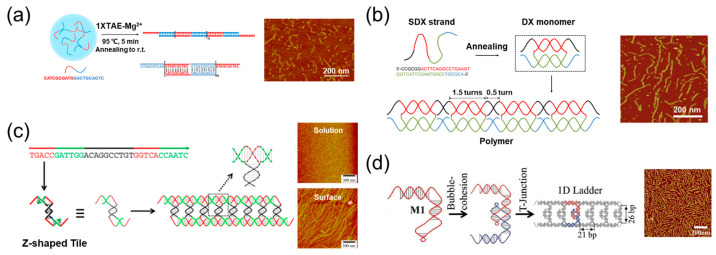
Some 1D assemblies using one DNA strand. (**a**) DNA supramolecular polymers with a DNA double-strand backbone [45]; (**b**) DNA supramolecular polymers with a DX backbone [47]; (**c**) DNA chains formed by a one-strand T-junction [48]; (**d**) 1D ladders assembled by one DNA strand via bubble cohesion and a T-junction [49].

The simplest system of 1D DNA assemblies is the long DNA double strand. In 2019, Liu et al. proposed a kinetically interlocking multiple-unit (KIMU) supramolecular polymer with a DNA double-strand backbone [45]. As illustrated in Figure 3a, the monomer was a 20-mer ssDNA (single-strand DNA) containing two different palindromic sequences. In the presence of Mg^2+^, the monomers could hybridize with each other to form long DNA double-strand polymers after annealing. Due to the cooperative effects of multiunit interactions, the polymer length was up to 72.7 nm and the polymer was kinetically stable even under very diluted conditions. In this study, it has been demonstrated that strong rigidity would encourage an increase in polymer length by suppressing cyclization. However, many nicks existed in the phosphate backbone in this KIMU polymer, decreasing the rigidity of the DNA double helix. Therefore, in order to construct longer polymers with the KIMU strategy, Liu et al. designed a DX monomer with a rigidity [46] that was nearly double that of the DNA duplex [47]. As shown in Figure 3b, a 44-mer ssDNA with four palindromic domains was synthesized. Two long palindromic domains (16 mer) were in the middle and two short palindromic domains (6 mer) were at both ends. In the annealing process, two long palindromic domains firstly hybridized to form a DX monomer, leaving the short palindromic domains outside as sticky ends. Then, the DX monomer was polymerized by the hybridization of two short palindromic domains. Due to the direction of the DNA double helix, the length of long and short palindromic sequences are key factors to determine the shape of assemblies. In this system, the length of long and short palindromic domains were approximately 1.5 and 0.5 turns of the DNA double helix, so there was no twist between the adjacent DX monomers and this ensured the formation of liner polymers. It has been demonstrated that the length of the polymer was nearly 2.5-fold that of the polymer with a DNA double-strand backbone, which was attributed to the increase in monomer rigidity.

In DNA assemblies, the Holliday junction is the most commonly used motif [50]. Recently, a novel T-junction [51] motif has been developed that can provide new geometry for DNA assembly [52]. Typically, T-junction motifs are composed of two different DNA single strands [53]. In 2014, Mao et al. utilized one DNA strand to form T-junctions that can assemble into 1D chains [48]. As illustrated in Figure 3c, the DNA strand contains five segments: a palindrome in the middle (black) and two pairs of complementary sequences (green and red) at the ends. Since the black palindrome is 1 turn of the DNA double helix, two strands would initially assemble into a Z-shaped tile by the hybridization of black and green segments. Four red segments were still in the single-stranded state and could further recognize each other among different tiles, resulting in the assembly of Z-shaped tiles and the formation of 1D chains eventually. AFM results illustrated that the chains assembled on the mica surface were significantly longer than assembled in the solution, which was caused by the decrease in entropy loss on the 2D surface that facilitates DNA assembly.

Generally, DNA duplex and hairpin structures [54] are two competitive products of the palindrome. Aforementioned assemblies are all based on a DNA duplex, requiring a long annealing time to prevent the formation of hairpin structures [55]. In 2017, Mao et al. proposed a new strategy for DNA assembly by making good use of a hairpin structure rather than fighting against them [49]. As shown in Figure 3d, a DNA single strand was rationally designed and could fold into hairpin structures by intramolecular hybridization. The hairpin structure contains two neighboring interior loops, one single-stranded bulge and one single-stranded overhang. Two hairpin structures could recognize each other and assemble into dimers via bubble cohesion of the two neighboring interior loops that are 6-mer palindromes. In this dimer, the vertical double helix domain was 2.5 turns so that the dimer was C shaped instead of Z shaped. Since the horizontal double helix domain was two turns, the C-shaped dimers would further assemble into 1D ladders by the hybridization of complementary bulge and overhang domains, which was verified by AFM images.

## 4. The 2D Materials Assembled by One DNA Strand

Almost all the 2D materials assembled by one DNA strand came from 1D materials. Taking advantage of the helical nature of a DNA duplex [56], the direction of DNA motifs could be regulated by simply changing base numbers [57], leading to the formation of 2D sheets rather than 1D chains.

**Figure 4:**
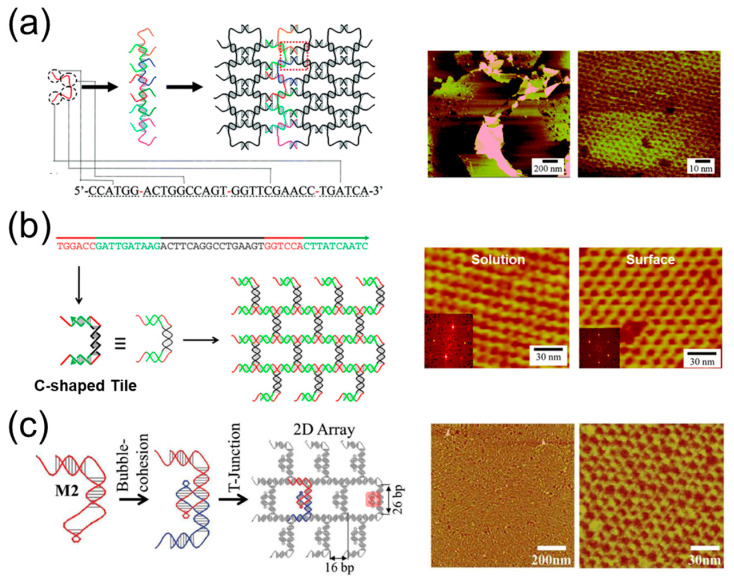
Some 2D assemblies using one DNA strand. (**a**) The 2D crystals formed by one DNA strand with four palindromes [58]; (**b**) 2D arrays assembled by a one-strand T-junction [48]; (**c**) 2D arrays formed by one DNA strand via bubble cohesion and a T-junction [49].

Among the reported 1D assemblies, a DNA strand with four palindromic domains can form supramolecular polymers. In 2007, Mao et al. used this system again to form DNA 2D crystals (Figure 4a) [58]. The DNA strand also contains four palindromic domains. In a previous study, the central long palindromic domains had 16 bases (1.5 turns). However, in this system, the long palindromic domain had 10 bases, equaling 1 turn of the DNA double helix. Therefore, instead of forming DX monomers, the DNA single strand would firstly self-assemble into long duplexes with 6-mer self-complementary overhangs every 10 base pairs. Then, with the decrease in annealing temperature, the long duplexes would form 2D crystals through hybridization of overhangs.

In addition, the systems in Figure 3c,d were also used to prepare 2D assemblies. In Figure 3c, the black and green domains had 10 and 6 bases, respectively. However, in the new system, the number increased to 16 and 10, indicating that both black and green domains have added a half turn of the DNA double helix, thus leading to the opposite direction of assemblies [48]. As shown in Figure 4b, the DNA strand would assemble into C-shaped tiles rather than Z-shaped tiles, and the final product was transformed from 1D chains to 2D arrays. Similarly, it was shown that the effect of assembly on the mica surface was much better than that in the solution. In Figure 3d, the vertical double helix domain was 2.5 turns, and the horizontal double helix domain was 2 turns. It was demonstrated that when the vertical domain remained unchanged and the horizontal domain was reduced to 1.5 turns, the C-shaped dimer would arrange differently (Figure 4c) [49]. Instead of hybridizing with other two dimers, the new C-shaped dimer would associate with another four dimers and form a branched structure, eventually resulting in the formation of 2D arrays.

## 5. The 3D Materials Assembled by One DNA Strand

The 3D assemblies can extend in any direction, and are much more sophisticated than 1D and 2D assemblies. For the construction of 3D assemblies, scientists can not only adjust DNA length to prepare different kinds of nanostructures, but also add the crosslinking domains to obtain microparticles and supramolecular hydrogels, which endow 3D assemblies with great potential for practical applications, such as drug delivery, biosensing and tissue engineering.

**Figure 5:**
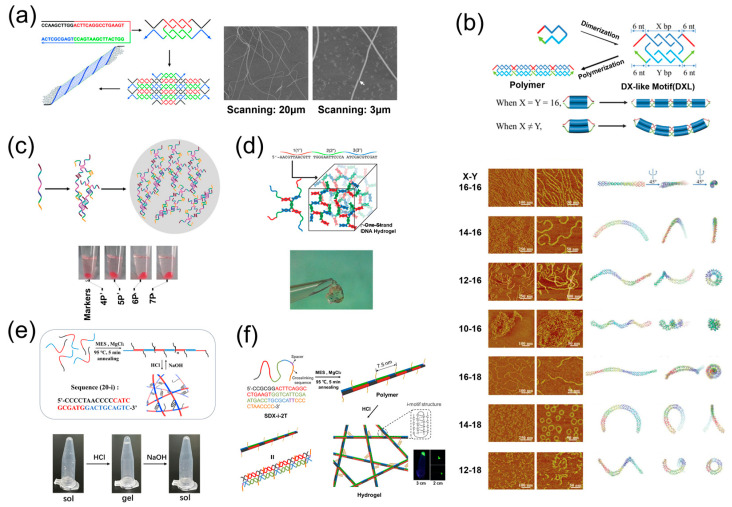
Some 3D assemblies using one DNA strand. (**a**) DNA nanotubes folded by 2D lattice assemblies [16]; (**b**) a series of DNA supramolecular polymers on adjusting the length [59]; (**c**) DNA microparticles through supramolecular homopolymerization of one DNA strand [60]; (**d**) DNA hydrogels assembled from multidomain DNA strands [61]; (**e**) pH-responsive DNA hydrogels with a DNA double-strand network [62]; (**f**) pH-responsive DNA hydrogels with a DX network [47].

Similar to Figure 3b, in 2006, Mao et al. prepared 3D nanotubes using a DNA strand with four palindromic domains but different lengths [16]. As shown in Figure 5a, the base number of four palindromic sequences was 10, 16, 16, 10, respectively. The length of long palindromic domains was the same as that in the previous system, but the length of short palindromic sequences was doubled—approximately 1 turn of the DNA double helix. Consequently, the adjacent DX monomers were arranged in the opposite direction rather than in the same line, leading to the formation of 2D lattices. Because there were many nicks in the lattices, they would fold into kinetically favorable nanotubes at low DNA concentrations. AFM images show that the nanotubes could reach 60 μm in length and 20–45 nm in diameter, and could be used as templates for nanofabrication. As mentioned above, a DNA strand with four palindromic domains can form 1D polymer chains, 2D crystals and 3D nanotubes. Although the length of the palindrome was different among these three systems, the length between the two long palindromes in each system was identical. In 2021, Mao et al. designed a series of supramolecular polymers with various morphologies by introducing differences in the length of the two long palindromes [59]. As shown in Figure 5b, they defined the base number of the two long palindromes as X and Y. When X = Y, the assembled DXL motifs were relaxed into a flat structure that could be polymerized into 1D chains. On the contrary, when X ≠ Y, there would be bending and twisting between two double helix structures in DXL motifs. During the process of polymerization, the bending and twisting would lead to the formation of spiral structures. A greater difference in length between two palindromes would bring higher bending and twisting in the DX monomer, resulting in greater curvature in the final products. In this study, a range of base numbers have been tested and corresponding nanostructures were generated, including straight 1D chains, spirals, and circles.

In addition to nanoscale structures, one DNA strand can also be used to prepare micron-sized particles. In 2019, Mao et al. reported a simple and novel strategy to prepare microparticles through the assembly of one DNA strand (Figure 5c) [60]. The DNA strand contained 4–7 palindromic domains and each domain was 10 bases in length. The adjacent domains were separated by two adenines to minimize steric hindrance, ensuring the occurrence of intermolecular hybridization rather than intramolecular hairpin structures. As each DNA strand would hybridize with multiple other strands, the final products were microparticles with crosslinked 3D networks. These microparticles could be further modified with functional domains and applied in capturing target molecules and stimulating immune responses.

Apart from the microscopic assemblies, macroscopic bulk materials, e.g., hydrogels, can also been manufactured utilizing one DNA strand. In 2016, Ke et al. proposed programmable DNA supramolecular hydrogels based on one DNA strand [61]. This was the first time that hydrogels were formed using one DNA strand. As shown in Figure 5d, the DNA strands contained 3–6 palindromic domains, which could assemble into branched structures and further crosslink to form 3D networks. Different numbers of domains would bring about different topological structures of 3D networks, leading to the regulation of thermal stability, pore size and mechanical properties of DNA hydrogels. Potential applications of hydrogels include loading and releasing cargo.

Responsive hydrogels can undergo rapid volume change and gel–sol transition under external stimuli, potentially expanding the application of hydrogels, for example, to sensing, controlled release and 3D printing. In 2017, Liu et al. reported pH-responsive supramolecular hydrogels assembled from one DNA strand (Figure 5e) [62]. The DNA strand contained two palindromic domains and a half i-motif domain. In alkaline conditions, the two palindromic domains hybridized to form polymer chains, leaving the half i-motif domain outside along the chains. When the pH decreased and the solution became acidic, the half i-motif domains could assemble into intact i-motif structures, leading to the crosslinking of polymer chains and the formation of hydrogels. The transition between the association and dissociation of the i-motif structure is reversible and fast, endowing the DNA supramolecular hydrogel with fast pH responsiveness. However, the backbone of this hydrogel is crosslinked DNA double strands with numerous nicks, with a rigidity that is relatively low, and this affects the mechanical strength and restricts the application of hydrogels. In order to solve this problem, Liu et al. replaced the DNA double helix backbone with a more rigid DNA double-crossover backbone to fabricate hydrogels [47]. As illustrated in Figure 5f, a 57-mer ssDNA was designed with four palindromic domains and a half i-motif domain at the 3′ end. After annealing, the four palindromic domains would hybridize to form DX polymer chains. According to the conformation, the half i-motif domains stretched out alternately on two sides of the chains. Under acidic conditions, 3D hydrogel networks could be formed by crosslinking i-motif structures. Here, a spacer with two thymine molecules was inserted between the palindromic domains and the half i-motif domains to eliminate the influence of steric hindrance on the formation of i-motif structures. Owing to the rigid DX backbone, the storage modulus of hydrogels was nearly 20 fold that of the hydrogels with the DNA double helix backbone.

## 6. The Principle for the Construction of DNA Materials Using One DNA Strand

After introducing the current reported systems from 1D to 3D, we summarized the principle using one DNA strand to construct materials and explained their assembly behavior using the principle. In the process of DNA assembly, DNA sequence, DNA length, buffer composition and annealing time are four major factors that influence assembly. Any minor changes may influence the morphology of the final products. Therefore, these four factors have to be precisely controlled to form defined structures.

### 6.1. The DNA Sequence

The DNA sequence is the most important factor that affects assembly. Generally, only the bases of two complementary DNA single strands can form a double strand through hybridization. This is the foundation for the construction of complicated structures. Therefore, to achieve DNA assembly using just one DNA strand, the sequences have to be palindromes [63]. In other words, the strand is self-complementary and can hybridize with itself. Nevertheless, the palindrome can also form intramolecular hairpin structures in the solution [64], commonly regarded as stoppers for the assembly. In some cases, hairpin structures can also be utilized to fabricate assemblies [49].

**Figure 6:**
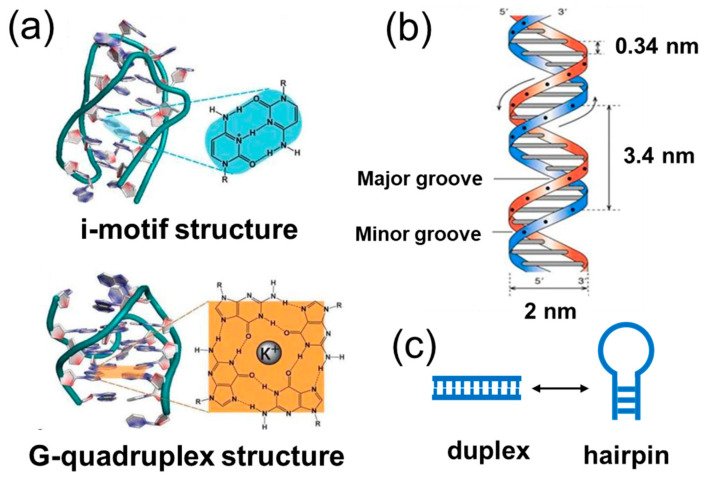
DNA secondary structures. (**a**) i-motif structure [65,66,67] and G-quadruplex structure [68,69,70]; (**b**) DNA double helix [6]; (**c**) equilibrium between duplex and hairpin structures.

Moreover, DNA strands with several special sequences can also assemble into quadruplex structures. As shown in Figure 6a, a sequence rich in cytosine can form an i-motif structure by combining a proton under weak acidic conditions [65,66,67]. A sequence rich in guanine can form a G-quadruplex structure through coordination in the presence of cations in the solution [68,69,70], such as K^+^ and Na^+^. Consequently, one DNA strand can also associate to form assemblies by making use of theses quadruplex structures.

### 6.2. DNA Length

In the classic Watson–Crick model, the DNA double helix has defined structures with a diameter of 2 nm (Figure 6b), and one turn of the DNA double helix contains 10.5 base pairs and the pitch is 3.4 nm [6]. Due to the helical nature of a DNA duplex, the length of the DNA strand can determine the direction and curvature of motifs, thus affecting the shape of assemblies. Additionally, DNA length can also influence the equilibrium between duplex and hairpin structures (Figure 6c) [71,72]. From the perspective of physical chemistry, a hairpin structure is kinetically favored and a duplex structure is thermodynamically stable. It can be anticipated that long DNA length could encourage the formation of hairpin structures and repress the assembly of duplex structures. Accordingly, it is crucial to design the DNA length to form 2D and 3D nanostructures.

### 6.3. Buffer Composition

Buffer composition is a key factor affecting DNA assembly, and the most significant factor of the buffer is the type and concentration of cations. Normally, compared with monovalent cations, divalent cations can effectively improve the interaction between bases and form more stable assemblies. Meanwhile, studies show that divalent cations can also promote the formation of duplex structures instead of hairpin structures [71].

### 6.4. Annealing Time

Annealing time mainly affects the kinetical behavior of the assembly, and further influences the assembled structures. A rapid annealing process can facilitate the formation of kinetically favored products, while a slow annealing process can form thermodynamically stable products. Since a duplex structure is much thermodynamically stable than a hairpin structure, it is necessary to prolong the annealing time, especially near the melting point, to promote the formation of duplex structures [71].

## 7. Conclusions and Perspectives

Owing to the specific base-pairing recognition, clear nanostructure, programmable sequence and responsiveness of the DNA molecule, DNA materials have attracted widespread attention in recent decades. In particular, preparation of DNA materials using just one DNA strand extremely simplifies the process of synthesis and assembly, greatly reducing the cost and improving efficiency. Multiple DNA strands are not required for this approach, thus preventing defects in final assemblies generated from the inaccuracy of DNA ratios. In this review, we summarized the reported materials from 1D to 3D using one DNA strand. The assembled materials not only included defined nanostructures but also contained microparticles and smart hydrogels, which could be used in controlled release, tissue engineering and 3D printing. We also discussed the principle for the construction of DNA materials using one DNA strand, including DNA sequence, DNA length, buffer conditions and annealing time.

Despite different kinds of materials assembled by one DNA strand having been demonstrated, there are still some crucial challenges to be resolved, particularly in the following fields: (1) Although the assembly approach using one DNA strand simplified the process, it also introduced many metastable structures that could compete with expected assembly structures. Some progress has already been made to avoid the metastable structures to some extents, but further research is still worth continuing to develop more effective control methods. (2) In current studies, Mg^2+^ is essential for assembly, decreasing the biocompatibility and restricting the clinical application of materials. More efforts should be made to develop biocompatible assembly systems. (3) In synthesis and purification, DNA may be assembled and crosslinked due to the hybridization of the palindrome, limiting the yield of the final products. The corresponding synthetic and purified methods need to be optimized.

In summary, DNA materials assembled by one DNA strand possess a lot of advantages compared with other traditional DNA materials. We believe that this approach will potentially pave the way to large-scale production and practical application of DNA materials.

## Data Availability

No new data were created or analyzed in this study. Data sharing is not applicable to this article.
